# Differential actions of indomethacin: clinical relevance in headache

**DOI:** 10.1097/j.pain.0000000000002032

**Published:** 2020-08-06

**Authors:** Oliver Summ, Anna P. Andreou, Simon Akerman, Philip R. Holland, Jan Hoffmann, Peter J. Goadsby

**Affiliations:** a Headache Group-Department of Neurology, University of California, San Francisco, San Francisco, CA, United States. Dr. Summ is now with the Department of Neurology and Research Center of Neurosensory Science, Carl von Ossietzky University Oldenburg, Oldenburg, Germany. Dr. Andreou is now with the Headache Research-Wolfson CARD, Institute of Psychiatry, Psychology and Neuroscience, King's College London, London, United Kingdom. Dr. Akerman is now with the Department of Neural and Pain Sciences, University of Maryland Baltimore, Baltimore, MD, United States; b Headache Group, Department of Basic and Clinical Neuroscience, Institute of Psychiatry, Psychology and Neuroscience, King's College London, London, United Kingdom

**Keywords:** Trigeminal, Nonsteroidal anti-inflammatory drugs, Cyclooxygenase inhibitor, Primary headache

## Abstract

Nonsteroidal anti-inflammatory drugs, cyclooxygenase inhibitors, are used routinely in the treatment of primary headache disorders. Indomethacin is unique in its use in the diagnosis and treatment of hemicrania continua and paroxysmal hemicrania. The mechanism of this specific action is not fully understood, although an interaction with nitric oxide (NO) signaling pathways has been suggested. Trigeminovascular neurons were activated by dural electrical stimulation, systemic administration of an NO donor, or local microiontophoresis of L-glutamate. Using electrophysiological techniques, we subsequently recorded the activation of trigeminovascular neurons and their responses to intravenous indomethacin, naproxen, and ibuprofen. Administration of indomethacin (5 mg·kg^−1^), ibuprofen (30 mg·kg^−1^), or naproxen (30 mg·kg^−1^) inhibited dural-evoked firing within the trigeminocervical complex with different temporal profiles. Similarly, both indomethacin and naproxen inhibited L-glutamate-evoked cell firing suggesting a common action. By contrast, only indomethacin was able to inhibit NO-induced firing. The differences in profile of effect of indomethacin may be fundamental to its ability to treat paroxysmal hemicrania and hemicrania continua. The data implicate NO-related signaling as a potential therapeutic approach to these disorders.

## Abstract 2

Indomethacin, naproxen, and ibuprofen inhibited nociceptive trigeminocervical neurons activated by stimulation of the dura mater, whereas only indomethacin inhibited responses activated by a nitric oxide donor.

## 1. Introduction

Primary headache disorders represent a substantial component of neurological practice. One important group is indomethacin-sensitive headaches, notably paroxysmal hemicrania and hemicrania continua.^27^ The question of what is unique about indomethacin compared to other nonsteroidal anti-inflammatory drugs (NSAIDs) is a crucial question for developing new therapies for these disorders.

Nonsteroidal anti-inflammatory drugs, which are cyclooxygenase (COX) inhibitors, are used in headache therapy, such as in migraine,^26^ in addition to their use in pain more broadly. They are distinguished by different chemical structures, eg, indomethacin is an acetic acid derivative, whereas ibuprofen is a propionic acid derivative; and by their profiles of absorption, metabolism, and excretion. Remarkably, NSAIDs do not act equally in all headache disorders. A particularly striking example is the indomethacin-sensitive trigeminal-autonomic cephalalgias: paroxysmal hemcrania^14,57^ and hemicrania continua.^13,58^ Indeed, the current diagnostic criteria for these disorders use an indomethacin response as a defining characteristic.^27^ An animal model recapitulating aspects of the trigeminal-autonomic cephalalgias has reported that indomethacin was significantly more effective than naproxen,^1^ suggesting an alternate mechanism of action from that previously demonstrated in animal models of migraine mechanisms.^6^ Taken together, it seems likely that indomethacin has a unique action that is not yet clarified.

Here, the effects of indomethacin, naproxen, and ibuprofen were compared in an established animal model of trigeminovascular nociception, which uses activation of dural nociceptive inputs in the trigeminocervical complex (TCC), believed to be important in the pathophysiology of a range of primary headache disorders.^25^ This model has proven effective at predicting antimigraine clinical efficacy.^6^ We also compared the local effects of microiontophoretically applied indomethacin and naproxen on iontophoresed glutamate-activated dural-responsive second-order neurons in the TCC. It has been suggested that indomethacin may modulate nitric oxide (NO) signaling pathways. Nitric oxide is known to induce headache and delayed migraine in patients.^2,32^ In preclinical studies, indomethacin is able to inhibit NO-induced dural vasodilation,^3^ and this effect is unique because naproxen or ibuprofen were ineffective in this regard.^61^ Nitrergic mechanisms may be involved in the pathophysiology of headache disorders and therefore indomethacin may target pathways of NO metabolism and signaling as its therapeutic action. We thus hypothesized that indomethacin may demonstrate differential modulation of second-order neurons with trigeminovascular nociceptive inputs and further that indomethacin may be differentially responsive at inhibiting NO-induced neuronal activity compared to naproxen or ibuprofen.

## 2. Materials and methods

All experiments were conducted under license of the University of California, San Francisco Institutional Animal Care and Use Committee and conforming to the National Institutes of Health Guide for the Care and Use of Laboratory Animals.^49^ Experiments adhered to the guidelines of the Committee for Research and Ethical Issues of the International Association for the Study of Pain^68^ and the ARRIVE guidelines.^35^

### 2.1. General

Male Sprague-Dawley rats (n = 65, 275-359 g) were used in all experiments, randomized to experimental groups, and analyzed by an observer blinded to their grouping. The selection was limited to male animals so that no interference of the estrous cycle was obtained and the number of animals used could be minimized for ethical reasons, while offering the opportunity of observing the effects of the tested drugs in steady conditions.^4^ Anesthesia was induced by intraperitoneal application of sodium pentobarbital (Nembutal, 60 mg·kg^−1^). After reaching a sufficient level of anesthesia, the animal was then placed on a thermostatically controlled homeothermic blanket and kept within physiological ranges. The femoral veins and left femoral artery were cannulated for intravenous administration of subsequent anesthesia, experimental drugs, and blood pressure monitoring (CT-1000 +ALM 932; CWE, Inc, Ardmore, PA). Anesthesia was maintained by intravenous application of sodium pentobarbital (25-30 mg·kg^−1^·h^−1^). The trachea was cannulated for mechanical ventilation with oxygen-enriched air (2-3 mL, 80-100 strokes·min^−1^, small rodent ventilator, Model 683; Harvard Instruments, Kent, United Kingdom). Adequate ventilation was monitored through end-tidal CO_2_ analysis (limit: 3.5%-4.5%, Capstar-100; CWE, Inc). For further procedures, animals were positioned in a stereotactic frame. The blood pressure, end-tidal CO_2_, and temperature were electronically displayed online, and together with the repeated observation of the animal's corneal and noxious withdrawal reflexes, used for monitoring suitable depth of anesthesia, and dose of sodium pentobarbitone was adjusted accordingly within the given range. Upon conclusion of electrophysiological recording protocols, all animals were euthanized by an i.v. dose of pentobarbital followed by central nervous tissue collection.

### 2.2. Electrophysiological recordings

For all electrophysiological recordings, a midline incision was made to expose the skull above the middle meningeal artery (MMA), and the appropriate area of the spine above the first and second cervical (C1/C2) levels. A small craniotomy above the MMA was then performed using a saline cooled dental drill, and a hemilaminectomy of C1 was performed, followed by a small incision of the dura mater so the recording electrode (either a 0.5 MΩ tungsten recording electrode, World Precision Instruments, United Kingdom, tip diameter 0.5 μm or microiontophoresis combination electrode: Carbostar 7s; Kation Scientific, Minneapolis, MN) could be lowered (piezoelectric motor/controller system: IW-811; Burleigh Instruments, Harpenden, United Kingdom; 8200 Controller; EXFO, Plano, TX) into the dorsal horn (5 µm steps) of the exposed TCC. Wide-dynamic-range neurons, identified by noxious pinch, and innocuous brush, responding to electrical stimulation of the MMA/dura mater (0.5 Hz, 0.1-0.2 ms, 5-16 V), were identified and recorded as described.^7^ For microiontophoretic experiments, the cells had to show stable baselines of increased firing rate in response to microiontophoretic L-glutamate ejections and stable baselines of increased firing rate in response to electrical stimulation of the MMA. The criteria for intravenous experiments did not include microiontophoretic L-glutamate ejections. Poststimulus histograms (PSTH) were established for sequences of 20 stimulations. A mean firing rate of 30% above baseline was required,^48^ within a 7 to 10 ms period of the main firing episode, corresponding to Aδ fibers. Poststimulus histograms were collected with 1-ms bin sizes over a poststimulus period of 100 ms. The action potential firing of the neurons recorded in response to microiontophoresis of L-glutamate were collected in successive 1-second bins and analyzed as cumulative rate histograms.

To study the intravenous effect of indomethacin, naproxen, and ibuprofen on electrical stimulation of the MMA, a baseline response was evaluated before administration of the drug/control baseline (mean out of 4 stimulation series of 20 sweeps). One of the tested drugs or the vehicle control (H_2_O for injection, pH 8-8.3) 1 ml·kg^−1^ was then administered intravenously. In all electrophysiological studies, animals received only a single dose of an NSAID intravenously. Further PSTHs were collected 5, 10, 15, 20, 25, 30, and 45 minutes after administration.

According to anatomical measurements and nerve conduction velocities, all recorded responses were meeting criteria for classification as A fibers (response 4-20 ms after stimulation).

All experimental and physiological data were acquired, displayed, and saved on a personal computer using an online data analysis system (Power 1401plus, CED and Spike5 software, United Kingdom).

At the end of the experiment, the recording site was electrically lesioned or marked by microiontophoretic ejection of pontamine sky blue (PSB), and tissue collected for further histological processing. Topological localization of lesion sites was then identified according to the Paxinos and Watson brain atlas.^52^

### 2.3. Microiontophoresis

By application of holding currents between 5 and 7 nA, with a polarity opposite to the charge of the respective ion, all ions were retained in the barrels.^60^ Ejection currents of the same polarity as the molecule's polarity were used for ejection of indomethacin and naproxen, and the charges ranged from 70 to 100 nA. The negative ejection currents for L-glutamate microiontophoresis ranged from 30 to 80 nA. To receive a response similar to receptive field stimulation of the first and second trigeminal dermatomes, the ejection current for L-glutamate was established individually for each recorded cell.

**Figure 1.**
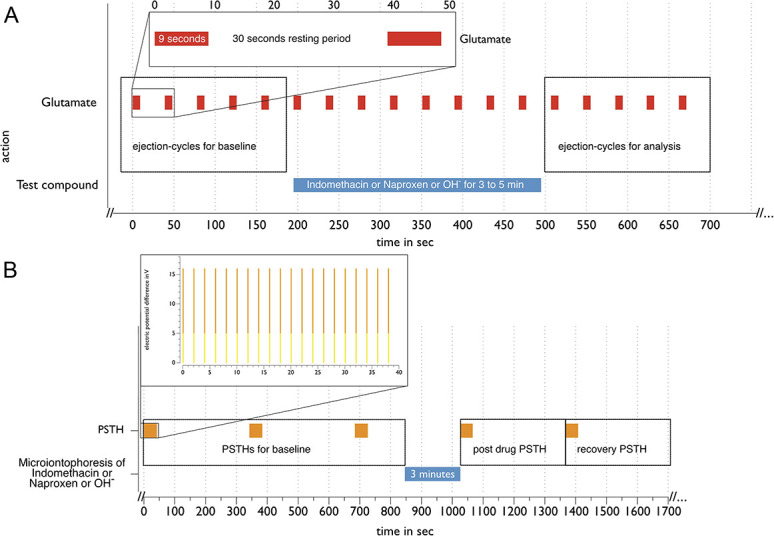
Timeline of microiontophoresis experiments. (A) One cycle of microiontophoresis establishing a baseline with repetitive L-Glutamate ejection-cycles, followed by microiontophoretic ejection of a test compound, and then an additional series. (B) One PSTH experiment, establishing a baseline with repetitive stimulation cycles of the MMA/dura for recordings followed by microiontophoretic ejection of one of the test compounds as indicated by horizontal bars. Further PSTHs are recorded at the time of termination of the ejection of the test compound, and a following PSTH during the recovery episode. The enlargement is displaying the timeline of a single PSTH recording, with the orange top of vertical bars indicating the range of voltage used for different experiments (5-16 V). The axis of abscissae displays time in sec within the timeline and enlargement. PSTH, poststimulus histograms.

To test the effect of indomethacin and naproxen on L-glutamate-evoked firing, L-glutamate was microiontophoresed, using a current generator (Dagan 6400; Dagan Corporation, MN), in ejection/retaining cycles with ejection period of 7 to 9 seconds and retaining period of 30 seconds of the ejection time to avoid desensitization of the L-glutamate response. Once 5 stable baseline responses were achieved, the testing compounds or control (OH^−^) were coejected over a period of 3 to 5 minutes of L-glutamate ejection, followed by recovery. For statistics, the first 5 cycles after the ejection of the test-compound ceased were analyzed (Fig. 1A).

For testing of the effect of the microiontophoresed compounds on MMA stimulation, a baseline response of 3 baseline PSTHs separated by five-minute recovery intervals was established. After further 2 minutes, this was followed by 3 minutes of microiontophoresis (−70 to −100 nA) of one of the drugs and a PSTH at the end of the microiontophoresis episode after further 5 minutes of recovery. After full recovery, this process was repeated with the other substances used (Fig. 1B). The drugs were given in pseudorandomized order. For full recovery, cells had to display evoked firing rates as before the application of the first compound tested and they were left for 30 minutes without further testing and establishing the according baseline. Resistances for the individual barrels ranged from 20 to 100 MΩ.

Pontamine sky blue was ejected (4 µA, 10 minutes) at the end of the experiment for later localization of the recording sites, and for reconstruction of further recording sites in compliance with the microdrive readings. After termination of each experiment, the brain tissue was collected and fixed in 10% formalin for histological processing.

### 2.4. Nitric oxide-induced trigeminal firing

In a separate group, all the animals received additional cannulation of the carotid artery, ipsilateral to the TCC recording site, for NO donor infusion. Having identified stable cells fulfilling the criteria for electrophysiological measurements: stable dural responsiveness and receptive field in the V1 branch of the trigeminal nerve, we observed the response of wide-dynamic-range neurons to intra-arterial administration of sodium nitroprusside (SNP), 2 µg·kg^−1^·min^−1^ dissolved in 0.9% saline solution, over 5 minutes. After a recovery period of 15 minutes, SNP infusion was repeated and the recorded firing rates of the duration of SNP infusion were averaged; the calculated value equals the SNP-induced activity at baseline. After a resting period of 3 minutes' control, indomethacin (5 mg·kg^−1^), naproxen (30 mg·kg^−1^), or ibuprofen (30 mg·kg^−1^) was slowly infused intravenously. Ten minutes after infusion, SNP injection cycles of 5-minute infusion time and recovery periods of 15 minutes were started. Sodium nitroprusside infusions were repeated at 10, 30, 50, and 70 minutes after drug administration.

### 2.5. Drugs

Microiontophoresis barrels of the combination electrode were filled with 200 mM L-glutamate monosodium, (Sigma, St. Louis, MO), pH 8.0; 15 mM indomethacin (Sigma), pH 8.0 to 8.3; 50 mM naproxen (Sigma), pH 8.0 to 8.3; distilled Water (dH_2_0), pH 8 to 8.5 as control; and 2.5% PSB (Gurr 6BX, BDH Laboratory Supplies, Poole, United Kingdom) in 100 mM sodium acetate and 200 mM NaCl for current balance.^8^ L-glutamate, indomethacin, naproxen, and PSB were ionized as anions. OH anions were microiontophoresed as control.

### 2.6. Data analysis

The experiments, recording the effect of the intravenous administered drugs on the electrically elicited firing rate in the TCC, were analyzed by comparison of the recorded PSTHs. The firing rate in response to SNP infusion was analyzed as the mean firing rate over 180 seconds pre-SNP infusion, and was subtracted from the mean firing rate during SNP infusion, resulting in the SNP induced firing rate. Analysis of the effect of microiontophoresed indomethacin and naproxen on L-glutamate-evoked firing was performed by calculation of the mean firing rate of 5 successive epochs of L-glutamate ejection predrug ejection. Testing of reliability was performed using Cronbach α. The mean response for each drug was then calculated by averaging the firing rate of 5 successive pulses during each drugs microejection. After ejection, 5 further pulses were averaged as the postejection response. The mean firing rate of spontaneous activity over 150 seconds was calculated and compared with the mean spontaneous firing during and after the microiontophoresis of each drug. For statistical analysis, we used IBM-SPSS (v20.0, New York, NY). The data sets of intravenous experiments and the effect of electrical stimulation and SNP, as well as microiontophoresis experiments (background and elicited activity) were analyzed by performing a mixed-model repeated-measures analysis of variance. Greenhouse–Geisser corrections were applied if the assumption of sphericity was violated. For all multiple comparisons, Bonferroni correction was applied. In the case of *P* values <0.05, post hoc comparisons were made using paired-sample *t* test for the effect of each intervention. The effect size *r* has been calculated using Pearson correlation coefficient. Results are expressed as percentages of baseline ± SE. Significance was assessed at the *P* < 0.05 level unless otherwise stated.

## 3. Results

### 3.1. General

Slow intravenous administration of vehicle control, indomethacin, naproxen, and ibuprofen had no effect on any physiological parameter recorded (cell firing rate at rest, blood pressure, and end-tidal CO_2_). The cells recorded in the experiments were identified being located within the TCC located in the dorsal horn within layers III-V at the level of C1.

### 3.2. Intravenous administration of test drugs

**Figure 2.**
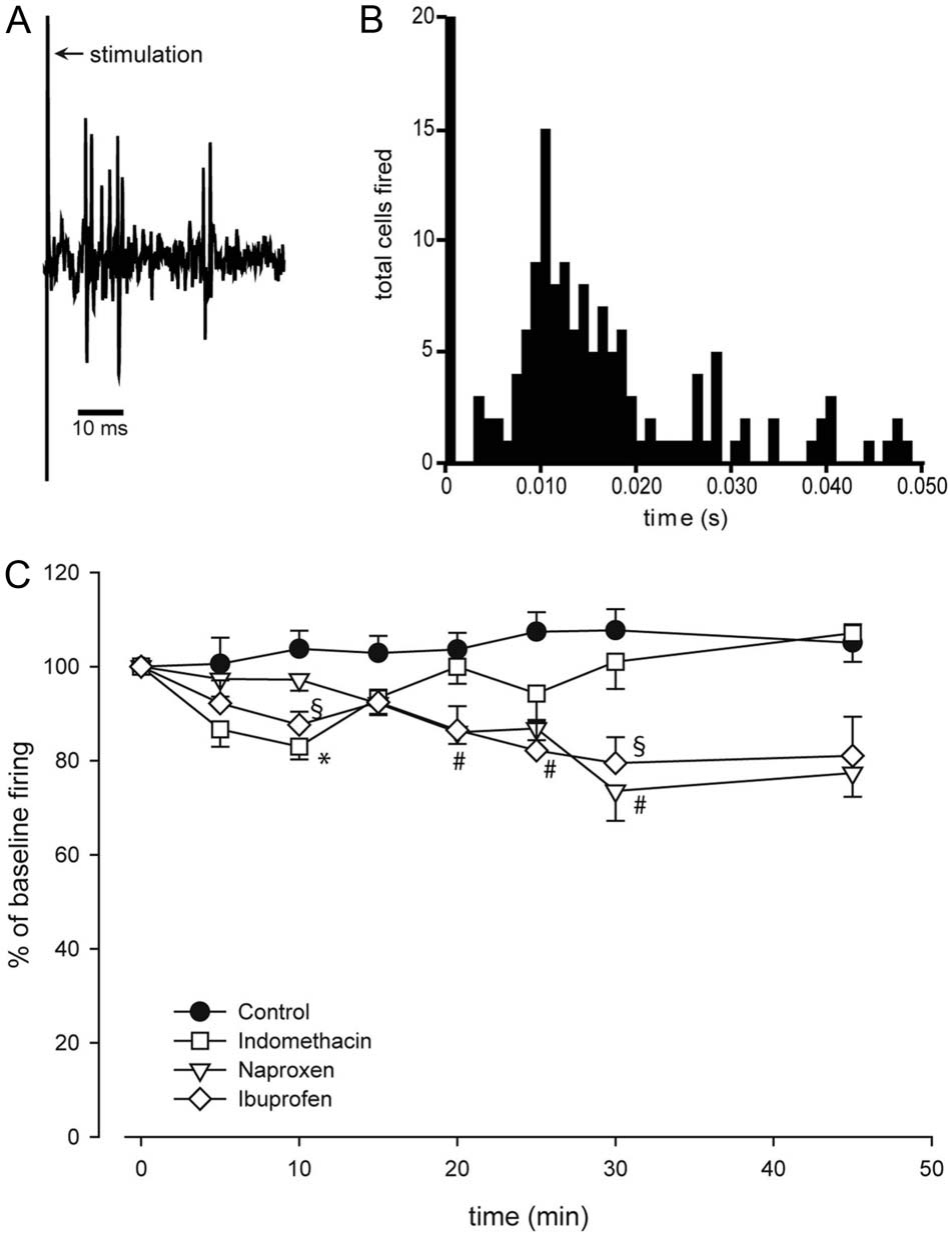
Display of responses to electrical stimulation of the dura mater/middle meningeal artery (A and B) and in relation to time after administration of indomethacin, ibuprofen, naproxen, and vehicle control (C). (A) Example single-unit trace of one of the recorded second-order neurons within the trigeminocervical complex, displaying the cluster response in reaction to electrical stimulation of the dura mater/middle meningeal artery. (B) Poststimulus histogram summarizing 20 responses of the second-order neuron shown in (A) (ordinate displaying the number of units firing due to electrical stimulation). (C) After the baseline response was established, one of the drugs (indomethacin 5 mg·kg^−1^, naproxen 30 mg·kg^−1^, ibuprofen 30 mg·kg^−1^, or control) was applied intravenously. All the tested drugs showed an inhibitory effect on the evoked firing with separate time point of maximum inhibition. Although indomethacin was demonstrated to induce early inhibition, naproxen induces a long-lasting inhibition at a later time point. Ibuprofen is shown to have early-onset inhibitory effect and a second inhibitory effect starting later and reaching its maximum parallel to naproxen (values for 0 indicate mean baseline responses). *, §, #*P* < 0.05 when compared to baseline response.

The effect of intravenously administered drugs on PSTHs recorded in the TCC was tested in 33 animals. Administration of indomethacin (n = 9; F_7,56_ = 4.072; *P* = 0.001) led to a 17 ± 3% inhibition of cell response 10 minutes after administration (*t*_8_ = 5.442, *P* = 0.001, *r* = 0.89) when compared to baseline. Naproxen (n = 8) demonstrated a slower profile, significantly inhibiting cell firing (F_2,14_ = 7.756; *P* = 0.006) after 20 to 30 minutes with a maximum inhibition of 26 ± 6% after 30 minutes (*t*_7_ = 3.828, *P* = 0.006, *r* = 0.82). Neuronal activation was also inhibited by ibuprofen (n = 8; F_7,49_ = 2.524; *P* = 0.027) over a prolonged period (10-30 minutes), with a maximum of 20 ± 5% 30 minutes after administration (*t*_7_ = 4.093, *P* = 0.005, *r* = 0.84; Fig. 2). Administration of vehicle control (n = 8) had no significant effect on neuronal activation (F_7,49_ = 0.738; *P* = 0.641).

### 3.3. Trigeminocervical complex microiontophoresis

The responses of 14 cells from 8 animals were investigated using the protocols for L-glutamate-evoked firing in conjunction with dural stimulation.

#### 3.3.1. L-Glutamate-evoked firing

**Figure 3.**
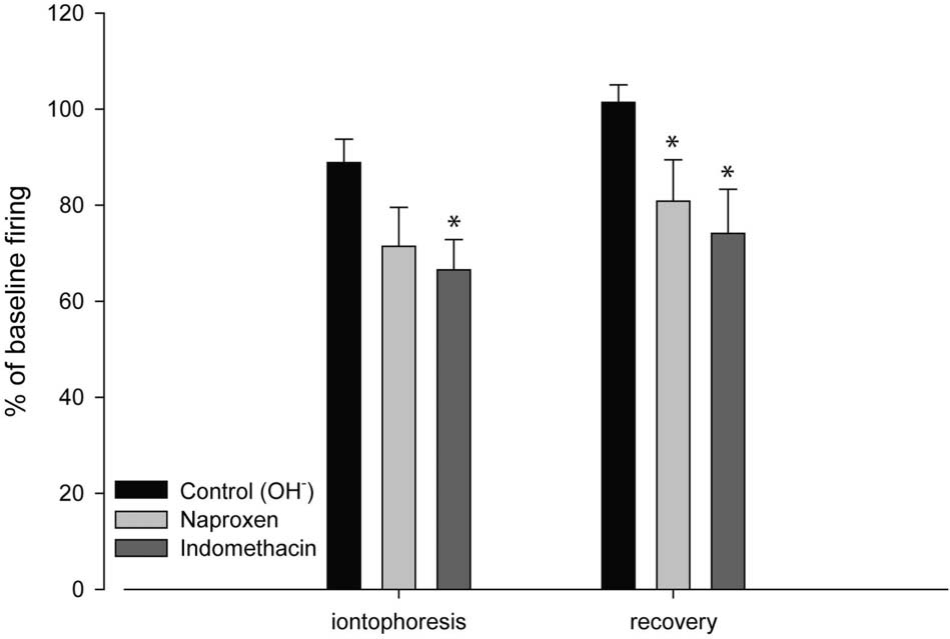
Effect of microiontophoresis of indomethacin, naproxen, and control on trigeminal neuronal firing elicited by repetitive microiontophoresis of L-glutamate. Summary of changes by microiontophoresis of indomethacin (−60 to 80 nA) vs naproxen vs control (OH^−^) at the same current. The bars representing the recovery period were calculated in the early recovery phase of the first 5 ejection cycles of L-glutamate after the ejection of the testing compound has ceased. *P < 0.05.

The current for microiontophoresis ranged from 60 to 80 nA, and drugs were microiontophoresed for 5 to 6 cycles with ejection periods of 5 to 10 seconds and retaining periods of 15 to 22 seconds. The baseline responses did not show significant differences (F_4,112_ = 1.401, *P* = 0.238) and were highly reliable (Cronbach α value ≥0.96). Application of control currents (cells n = 8) had no effect on L-glutamate-evoked cell firing in the TCC (F_2,16_ = 1.739, *P* = 0.207). Indomethacin (cells n = 9) significantly inhibited neuronal activity (F_2,16_ = 8.123, *P* = 0.004) by 22 ± 8% (*t*_15_ = 2.739, *P* < 0.015, *r* = 0.58) compared to control. After ejection ceased, L-glutamate cycles continued to be inhibited by 27 ± 10% (*t*_10_ = 2.748, *P* < 0.020, *r* = 0.66). This effect lasted for up to 15 minutes. Naproxen (cells n = 7) also showed an effect on L-glutamate-evoked cell firing in the TCC (F_2,12_ = 10.525, *P* = 0.002). However, the onset of the effect was not significant during the ejection of the drug but during the recovery period, displaying a 21 ± 9% (*t*_13_ = 2.298, *P* = 0.039, *r* = 0.54) inhibition (Fig. 3). The microiontophoresis of control ions, indomethacin, or naproxen had no significant effect on the spontaneous neuronal activity in the TCC (all *P* ≥ 0.28).

#### 3.3.2. Dural stimulation and microiontophoresis

The microiontophoresis of control ions, indomethacin, or naproxen had no effect on neuronal firing due to electrical stimulation of the dura mater (cells n = 11; *P* ≥ 0.27).

### 3.4. Sodium nitroprusside infusion induced activity

**Figure 4.**
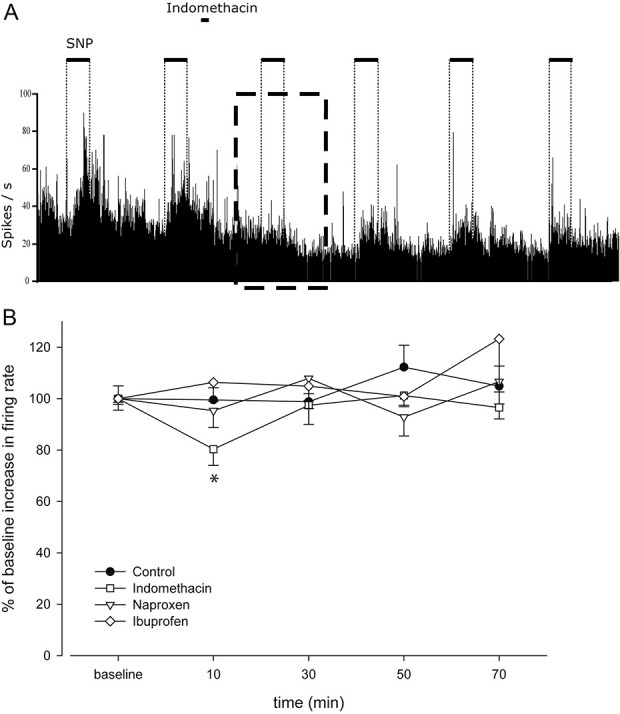
Representative recordings of firing rates (A) in the trigeminocervical complex from experiments investigating the effect of indomethacin on second-order neuronal activity, induced by repeated sodium nitroprusside (SNP) infusions. The neuronal activity increases shortly after SNP (2 µg·kg^−1^·min^−1^) administration. The box in A indicates the decreased neuronal response due to infusion of SNP 10 minutes after intravenous administration of indomethacin. After the baseline response was established, one of the drugs (indomethacin 5 mg·kg^−1^, naproxen 30 mg·kg^−1^, ibuprofen 30 mg·kg^−1^, or control) was applied intravenously, followed by SNP infusions (each for a 5-minute duration, followed by a 15-minute recovery period, starting 10 minutes after the administration of one of the drugs). An inhibitory effect on the SNP-induced firing was registered exclusively for indomethacin (B). *P < 0.05 when compared to baseline response.

The SNP protocol was conducted in 24 animals. The infusion of SNP increased the background firing rate of the tested cells (t_23_ = 8.775; *P* = 0.000, *r* = 0.88) by 26 ± 3%, returning to baseline levels within 2 minutes after each SNP infusion (Fig. 4A). The background activity between SNP infusions remained unchanged in the absence of drug treatment throughout the experiments, and no changes in the expansion of dural and cutaneous receptive fields were observed. Intravenous administration of indomethacin significantly altered TCC activity induced by SNP infusion (F_4,20_ = 3.19, *P* = 0.035; Figs. 4A and B). By contrast, control (F_2,11_ = 1.24, *P* = 0.331), naproxen (F_4,20_ = 0.67, *P* = 0.62), or ibuprofen (F_1,7_ = 1.12, *P* = 0.354) did not alter the increase of background activity in response to SNP infusions (n = 6 per group; Fig. 4B).

In line with results of a previous study, we monitored a minor drop in the baseline blood pressure when SNP infusion was started, and the reading returned to baseline level within 3 minutes after SNP infusion.

## 4. Discussion

The current study provides evidence of a substantial difference of the tested NSAIDs' capabilities for blocking the SNP infusion-induced effects in the TCC. When comparing the effects of the NSAIDs on electrically induced cell firing within the TCC, it becomes clear that they have individual time-dependent profiles of action. Here, we demonstrate a transient inhibitory effect after application of indomethacin on NO-induced, as well as electrically induced trigeminal firing in the TCC, that is evident only 10 minutes after drug infusion, whereas the inhibitory effect on NO-induced vasodilation has been shown to outlast 70 minutes.^61^ In line with the effects of naproxen on electrically induced vasodilation, it had a much slower onset of effect on electrically induced firing in the TCC and reached its peak inhibition near the end of the experiment, a possible sign of a delayed passage of the blood–brain barrier as has been described earlier.^21,22^ In a similar in vivo model, acetylsalicylic acid and ketorolac, which is structurally related to indomethacin, were demonstrated to have a time course of inhibition matching the one of naproxen measured in our experiments,^34^ although these experiments were conducted in cat.

The unique effects of indomethacin demonstrating inhibitory modulation of NO-induced vasodilation and trigeminal activity, with a rapid onset, do not define whether this is a central or peripheral effect, although experimental data suggest a limited penetration through the blood–brain barrier.^64^ A significant central action of indomethacin is supported by the diversity in the time course of indomethacin's effect in this and our previous experiments. The recorded activity after SNP infusion is unlikely to result from peripheral vascular effects over 70 minutes.^61^ Because hemicrania continua is a long-lasting headache, it remains unclear, however, to what extent the central NO-activated mechanisms investigated here are relevant to hemicrania continua and paroxysmal hemicrania and if it is the central or peripheral NO-induced effect. As stated before, indomethacin elicited a clear potential in inhibiting dural-evoked activation and interestingly a long-lasting inhibitory effect on the activation recorded in the TCC after stimulation of the superior salivatory nucleus.^1^ Combining these results involvement of NO mechanisms, modifiable by indomethacin, at the level of the superior salivatory nucleus is a possible mechanism. Indomethacin may activate modulatory mechanisms within the central nervous system not investigated here. Interestingly, indomethacin can modulate nociceptive signaling in a model of trigeminal autonomic cephalalgias,^1^ which is in line with the data presented here.

The dose of indomethacin, ibuprofen, and naproxen used was based on the knowledge of similar high absorption rates and plasma protein binding if taken orally, as well as the ratio of their maximum daily dose for the treatment of headache, *viz*., indomethacin: ibuprofen: naproxen = >225 mg·d^−1^:1200 mg·d^−1^:1200 mg·d^−1^ = 1:6:6. We used the same doses as previously reported^61^ because these doses have been shown to be well tolerated; the dose for indomethacin is slightly lower than its oral LD_10_.^50^ There is a broad knowledge from in vitro experiments about NSAIDs' action on the different cyclooxygenases, including their kinetic profiles^23^ and their capabilities in the inhibition of COX1/COX2 mechanisms, resulting in specific COX1/COX2 ratios.^45^ COX1 and COX2 are prominent, not only in the dura mater as a key structure of trigeminal innervation,^42,67^ but COX2 activity has been shown to be modifiable at the level of the caudal trigeminal trigeminocervical nucleus.^65^ COX1/COX2 inhibition has been shown to alter trigeminocervical neurons responding to nociceptive dural activation.^34^ Interestingly, the inhibitory effect of the tested drugs at the level of the TCC seems to be rather limited compared to the effect of triptans, such as rizatriptan and naratriptan.^15,16^ This is in contrast to the greater use of NSAIDs in migraine, although it is consistent with severe migraine attacks being generally better treated with triptans. Certainly, in clinical trials, fewer patients respond to NSAIDs than triptans.^10^

The microiontophoretic data demonstrate a central effect of indomethacin and naproxen on postsynaptic second-order neurons within the TCC. However, in our experimental setup, no effect was seen when we studied the microiontophoretic modulation of TCC firing. This might be due to a comparatively low potency of the tested drugs in the TCC. The effect of ibuprofen was not investigated microiontophoretically for technical reasons regarding the electrical properties of the compound.^54^

Investigating the interaction of indomethacin with NO and NO-mediated mechanisms, indomethacin has been shown to reduce NO production from rat microglia.^17^ Indomethacin also inhibits the expression of endothelial NO synthase in vivo measured in the kidneys^47^ and inducible NO synthase (iNOS) in macrophages in vitro,^30^ a mechanism that is most likely due to decreased PGE2 production through COX2, as it has been shown that PGE2 is able to upregulate iNOS expression in macrophages in vitro.^11,51^ Yet, this mechanism is unlikely to be essential for our results because SNP is a direct donor of NO without iNOS involvement and the selective iNOS inhibitor GW274150 failed in prophylactic treatment of migraine.^28^ The NO donor glyceryl trinitrate has been known to trigger migraine for some time.^29^ The delayed headache has the typical clinical phenotype of a migraine attack.^2,31,55^ Glyceryl trinitrate is also capable of triggering a cluster headache attack with a delay of about 10 minutes after infusion, if it is used during an active cluster period.^20^ Given this context, the modulation of NO-induced early activation of trigeminal activity, as demonstrated here, suggests that NO might play a significant role in the pathophysiology of paroxysmal hemicrania and hemicrania continua. Interestingly, NO also demonstrated modulatory effects through activation of Na_v_1.9 channels in a mouse model of triptan-induced medication overuse headache,^9^ and indeed indomethacin has been reported to produce a migraine-like headache when used in hemicrania continua.^33^

Nitric oxide release facilitates the parasympathetic craniofacial pathways^24^ as one key structure within the pathophysiological pathway of trigeminal autonomic cephalagias.^44^ Nitric oxide donors have previously been described to cause activation of neurons within the spinal trigeminal nucleus, midbrain, and forebrain structures when tested in animal studies.^53,62^ Direct activation of trigeminovascular neurons as well as sensitization was found performing electrophysiological work using GTN in an animal in vivo study.^2,39^ This suggests the activation, driven by SNP infusion, may be at least partly caused by local NO effects in the TCC or other central structures with modulatory connectivity to the TCC. This includes cervical inputs^5^ and structures feeding the downstream modulatory pathway to the TCC such as the periaqueductal gray,^36^ rostral ventromedial medulla,^19^ and hypothalamic nuclei such as the A11.^12,59^ Nitric oxide is known to activate cells through indirect elevation of cyclic guanosine-monophosphate (cGMP) levels,^46^ causing vasodilation through subsequent decrease of Ca^2+^. Independent from cGMP levels, it is also capable of modulating the level of CGRP release within the trigeminal ganglion.^18^ In addition to these effects, the microiontophoresis of an NOS inhibitor at the level of the TCC showed inhibitory effects on L-glutamate and electrical-induced activity of second-order neurons, thereby demonstrating a direct effect on second-order neurons.^40^ Nitric oxide/nNOS activity has also been suggestive of antinociceptive capabilities in chronic inflammation,^63^ yet these effects are limited to chronic pain and rely on nNOS activity.

In our experiments, there was no sensitization as determined by altered baseline activity, change of dural, or cutaneous receptive fields. Although NO has been described to induce central sensitization in a rat model, infusion of SNP intravenously did not produce a significant immediate facilitation of trigeminal firing.^37^ Nitric oxide is a highly volatile molecule with a very short half-life^46^; so, we may have achieved higher levels of NO at relevant structures, although the dose of SNP per injection period was lower in our experimental setup (10 vs 50 µg·kg^−1^), when compared to that of Koulchitsky et al,^37^ and higher than in their study where they investigated sensitization at the level of trigeminal nucleus.^38^ A comparison of these studies is, however, challenging because SNP was infused into the carotid artery ipsilateral to the electrophysiological recording sites, with similar direct action to what has been reported.^39^ Studies of the effect of NO donors on neuronal activity in the trigeminal ganglion, but not the TCC, found a delayed activation.^41,66^ This might also be attributed to an increase in local CGRP levels, as well as the receptor activity modulating protein 1 (RAMP-1) component of the CGRP receptor because it was immunohistochemically demonstrated after i.v. administration of GTN to rats.^56^ Application of CGRP itself on trigeminal ganglia cultures, however, demonstrated increased iNOS expression and NO release.^43^

In summary, the data, taken with previous work, suggest a possible central action of the NSAIDs indomethacin and naproxen. An inhibitory effect on electrically induced trigeminal firing is demonstrated for indomethacin, ibuprofen, and naproxen; by contrast, the effect on NO-evoked trigeminal firing can be seen for only indomethacin. The results offer new insights into NSAID mechanisms in primary headache disorders and highlight alternative signaling pathways involved in the particular pathophysiology of hemicrania continua and paroxysmal hemicrania. Moreover, the data are consistent with the substantially central nervous system pathogenesis of paroxysmal hemicrania and hemicrania continua. Taken together with our previous studies, perhaps therapies directed at nitrergic mechanisms may be a promising target for the treatment of paroxysmal hemicrania and hemicrania continua.

## Conflict of interest statement

O. Summ has no conflicts of interest to declare; and reports a fellowship from MSD Sharp & Dohme GmbH not related to the work. A.P. Andreou declares no direct conflicts; and reports equipment grant from eNeura, honoraria and travel expenses from Eli Lilly and eNeura, in relation to educational duties and advisory boards, as well as sponsorships for educational purposes from Autonomic Technologies, eNeura, Allergan, Eli Lilly, and Novartis. S. Akerman declares no conflicts; and reports personal fees from Allergan, Amgen, GSK, Novartis, and A&O unrelated to the submitted work. P.R. Holland declares no direct conflicts; and reports research grants from Amgen, Celgene, and Eli Lilly, as well as honoraria and travel expenses in relation to educational duties and advisory boards from Allergan, Novartis, and Almirall. J. Hoffmann declares no direct conflicts and reports honoraria for consulting activities and/or serving on advisory boards from Allergan, Autonomic Technologies Inc, Chordate Medical AB, Eli Lilly, Hormosan Pharma, Novartis, and Teva. He received personal fees for medicolegal work as well as from Sage Publishing, Springer Healthcare, and Quintessence Publishing. All these activities are unrelated to the submitted work. J. Hoffmann reports a research grant from Celgene. P.J. Goadsby declares no direct conflicts, and reports, over the last 36 months, grants and personal fees from Amgen and Eli-Lilly and Company, grant from Celgene, and personal fees from Alder Biopharmaceuticals, Allergan, Aeon Bopharma, Biohaven Pharmaceuticals Inc, Clexio, Electrocore LLC, eNeura, Epalex, Impel Neuropharma, MundiPharma, Novartis, Santara Therapeutics, Teva Pharmaceuticals, Trigemina Inc, and WL Gore, and personal fees from medicolegal work, Massachusetts Medical Society, Up-to-Date, Oxford University Press, and Wolters Kluwer; and a patent magnetic stimulation for headache assigned to eNeura without fee.
